# Novel Generalized Low-Pass Filter with Adjustable Parameters of Exponential-Type Forgetting and Its Application to ECG Signal

**DOI:** 10.3390/s22228740

**Published:** 2022-11-12

**Authors:** Ivo Petráš

**Affiliations:** Faculty of BERG, Technical University of Košice, Němcovej 3, 042 00 Košice, Slovakia; ivo.petras@tuke.sk; Tel.: +421-55-602-5194

**Keywords:** denoising, ECG, exponential-type forgetting, Gaussian function, Gaussian filter, Mittag–Leffler function, Mittag–Leffler filter

## Abstract

In this paper, a novel form of the Gaussian filter, the Mittag–Leffler filter is presented. This new filter uses the Mittag–Leffler function in the probability-density function. Such Mittag–Leffler distribution is used in the convolution kernel of the filter. The filter has three parameters that may adjust the curve shape due to the filter-forgetting factor. Illustrative examples present the main advantages of the proposed filter compared to classical Gaussian filtering techniques, as well as real ECG-signal denoising. Some implementation notes, along with the Matlab function, are also presented.

## 1. Introduction

Filtering is processing a signal whereby some unwanted components or properties are removed from the signal, or some aspects of the signal are suppressed. It often means removing some frequencies or frequency bands from the signal. However, we can use filters sparingly in the frequency domain, and specific frequency components can be removed without having to act in the frequency domain. Filters are widely used in various areas, for example, in signal processing in electronics and telecommunications, radars, control systems sensors, image processing, and computer graphics. Different forms of filters are often used, for instance, the Laplacian filter [1], Bayesian filter [2], Gaussian filter [3], and so on. A significant field of applications is filtering signals from biomedical systems sensors, for instance, electroencephalographic (EEG), electromyographic (EMG), or electrocardiographic (ECG) signals. Many denoising methods exist for such types of bio-signals, which are described in the literature, e.g., [4,5,6].

This paper describes a new filter based on the classical Gaussian filter. It is well known that this filter is often used in signal- and image-processing areas for smoothing and noise reduction, e.g., [7,8,9], including ECG signals [10,11,12].

It is known that heart-related problems can be identified and detected using ECG signals, which are popular and extensively used as a diagnostic tool for revealing related diseases. Decision-making or classification accuracy entirely depends on ECG signal quality. Filtering the noise-signal components is a highly challenging problem.

Considering that the famous Gaussian filter is also useful for ECG signals, the idea for a novel generalized low-pass filter, which would be better than the Gaussian filter and have a similar structure, came.

The Gaussian filter is a convolutional filter that uses a Gaussian function as a convolution kernel and mathematically adjusts the input signal by convolution with a Gaussian function. In other words, a Gaussian filter is a filter whose impulse response is a Gaussian function. Among other things, these filters have the important property that they do not overshoot at the input of the step function and, at the same time, minimize the rise and fall time. This behavior is closely related to the Gaussian filter having the minimum possible group delay.

The main contributions of this paper are as follows:

- The generalization of the classical Gaussian filter to the novel form, based on the Mittag–Leffler distribution function,
- The suggestion of the implementation algorithm for the new Mittag–Leffler filter with adjustable forgetting parameters,
- The support of proposed algorithm in the form of the Matlab function.

The structure of this paper is as follows. Section 1 briefly describes the introduction to the problem. Section 2 presents the essential mathematical tools and methods. The main results are shown in Section 3 to demonstrate the benefits of the proposed new filter. Finally, some concluding remarks are given in Section 4.

## 2. Methods

### 2.1. Gaussian Function and Gaussian Distribution

The Gaussian function, named after Johann Carl Friedrich Gauss, is a function that can be expressed in elemental form

(1) $$f\left(x\right)=ae^{-\frac{{\left(x-b\right)}^{2}}{2c^{2}}},$$

for arbitrary real parameters *a*, *b*, and c>0.

Gaussian functions (1) are often used in statistics to represent the probability-density function (PDF) of a normal shifted distribution (a.k.a. Gauss distribution) for a real-valued random variable with the expected value (or mean) b=μ and variance $c^{2}=\sigma^{2}$. The general form of its PDF ϕ(x) is

(2) $$\phi \left(x;\sigma \right)=\frac{1}{\sigma \sqrt{2\pi }}e^{-\frac{1}{2}{\left(\frac{x-\mu }{\sigma }\right)}^{2}},$$

where the variable μ∈R is the mean (or expectation) of the distribution, while the positive variable σ∈R is its standard deviation. The variance of this Gauss distribution is then $\sigma^{2}$.

The simplest case of the normal distribution is known as the normal unit distribution or the standard normal distribution. This is a particular case when σ=1 and μ=0. It means that *x* has variance, a standard deviation of 1, and a mean of 0.

Except for the mentioned utilization of the Gaussian function as PDF for normal distribution, we may use it in signal processing to define Gaussian filters and image processing, where a two-dimensional Gaussian filter is used for blurs.

Moreover, the exponential law is the classical approach to studying the dynamics of systems, but there are many systems where dynamics obey a faster or slower law than the exponential law. In that case, the Mittag–Leffler function can best describe such anomalous dynamics changes [13].

### 2.2. Mittag–Leffler Function and Mittag–Leffler Distribution

The Mittag–Leffler function $E_{\alpha ,\beta }\left(z\right)$, named after Magnus Gustaf Mittag–Leffler, is a special function that depends on two parameters, α and β. It may be expressed by the following series [14]:

(3) $$E_{\alpha ,\beta }\left(z\right)=\sum_{n=0}^{\infty }\frac{z^{n}}{\Gamma (\alpha n+\beta )},\;\alpha ,\beta >0,\;\;z\in C,$$

where Γ(.) is the gamma function, for β=1, we obtain a one-parameter Mittag–Leffler function $E_{\alpha ,1}\left(z\right)\equiv E_{\alpha }\left(z\right)$.

The Mittag–Leffler function is sometimes called the queen of functions [15]. There are relations between this function and other functions, for instance,

(4) $$E_{1,1}\left(z\right)=e^{z},\;\;E_{0^{+},1}\left(-z^{2}\right)=\frac{1}{1+z^{2}},\;\;E_{2,1}\left(-z^{2}\right)=\cos\left(z\right).$$

The Mittag–Leffler function appears naturally in the solution of fractional differential equations, which is essential in fractional calculus theory [14]. The ordinary and generalized Mittag–Leffler functions interpolate between a purely exponential-law and power-law behavior. It is an important property that may be used in a filter with variable exponential forgetting. However, there are methods where fractional calculus (fractional-order derivatives/integrals) can be directly used in filter design and signal processing [16,17,18].

**Figure 1:**
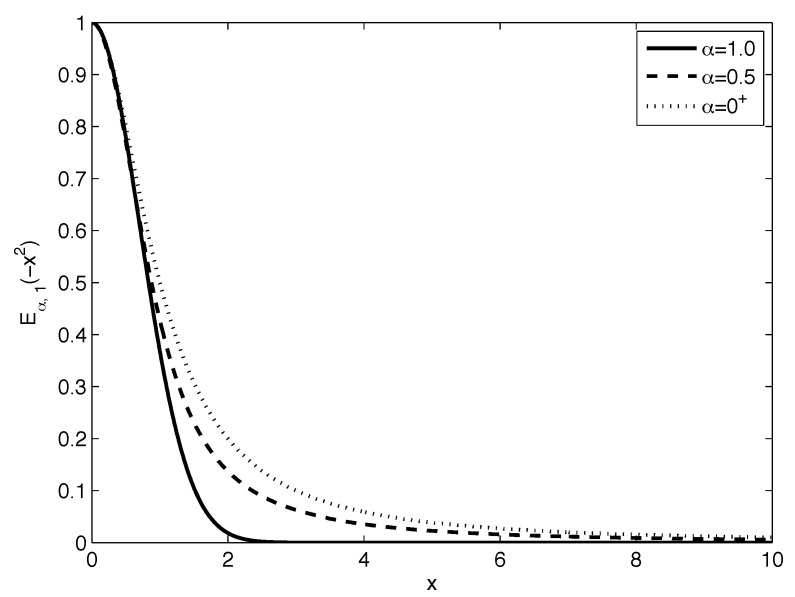
Behaviour of the Mittag–Leffler function $E_{\alpha ,1}\left(-x^{2}\right)$ for various parameters α within interval α∈(0;1] and fixed β=1.

**Figure 2:**
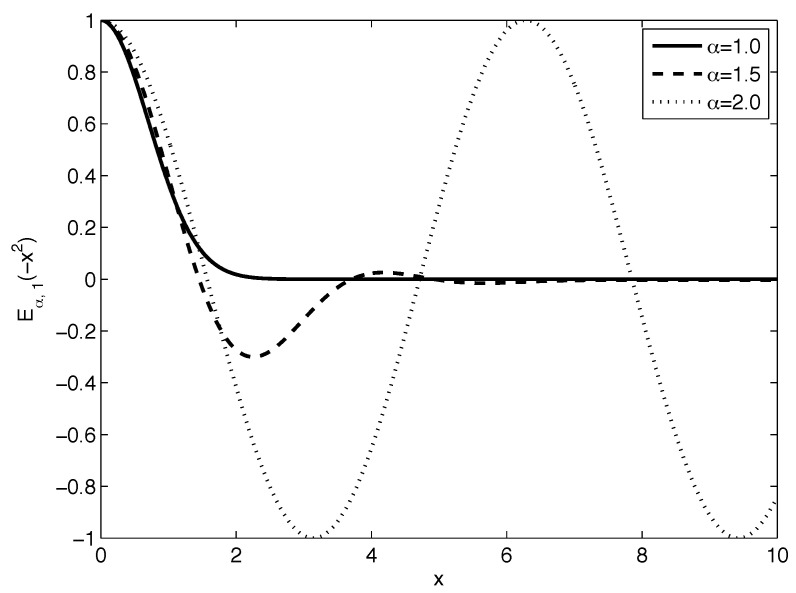
Behavior of the Mittag–Leffler function $E_{\alpha ,1}\left(-x^{2}\right)$ for various parameters α within interval α∈[1;2] and fixed β=1.

Figure 1 and Figure 2 plot the Mittag–Leffler function $E_{\alpha ,1}\left(-x^{2}\right)$ behavior for various values of α and β=1, respectively.

Furthermore, the Mittag–Leffler distribution with parameter α proposed in [19] can be written by the PDF as

(5) $$\phi \left(x;\sigma ,\alpha \right)=\frac{\sqrt{\sigma }}{\pi }\Gamma \left(\frac{\alpha }{2}\right)E_{\alpha ,\alpha }\left(-\sigma {\left(x-\mu \right)}^{2}\right),$$

where 0<α≤1,  σ>0, and μ∈R. Earlier, in 1990, it was proved by Pillai [20] that the Mittag–Leffler distribution with parameter α is attracted to the stable distribution only with exponent α, 0<α≤1. It is related to the behavior of the Mittag–Leffler function depicted in Figure 1. Hence, function $1-E_{\alpha ,1}\left(-x^{\alpha }\right)$ is the cumulative distribution function of a probability measure on the non-negative real numbers.

A Mittag–Leffler distribution with parameters α and β characterized by the following PDF was considered in [19]:

(6) $$\phi \left(x;\sigma ,\alpha ,\beta \right)=\frac{\sqrt{\sigma }}{\pi }\Gamma \left(\beta -\frac{\alpha }{2}\right)E_{\alpha ,\beta }\left(-\sigma {\left(x-\mu \right)}^{2}\right),$$

where 0<α≤1,  σ>0, μ∈R, and β≥α in (6). For α=β, we obtain the distribution described by (5). Some other important properties and relations to stochastic processes can be found in [19,20,21,22].

However, as we may observe in Figure 2, the behavior of the Mittag–Leffler function for parameter α>1 leads to oscillations, and therefore such distribution is related to a negative probability. Several authors have already observed such behavior, for instance, in 1942 Paul Dirac [23] and in 1987 Richard Feynman [24]. Recently, Leonenko and Podlubny have shown that the extension of the Monte Carlo approach to the fractional differentiation of orders higher than one led to working with signed probabilities that are not necessarily positive [25]. We adopted this idea for further consideration, and here we define the following more appropriate Mittag–Leffler distribution by the following PDF:

(7) $$\phi \left(x;\sigma ,\alpha ,\beta \right)=\frac{1}{\sigma \sqrt{2\pi }}E_{\alpha ,\beta }\left(-\frac{{\left(x-\mu \right)}^{2}}{2\sigma^{2}}\right),$$

where σ, α, and β are positive filter parameters with 0<α≤2,  0<β≤2, and μ∈R is the mean value of an independent variable *x* usually denoted by symbol $\bar{x}$.

It should also be noted that the Mittag–Leffler distribution is connected to related and other families of distributions [26].

### 2.3. Problem Formulation

In general, the objective of the filter is to extract a true signal from the noisy measured signal

(8) $$y\left(t\right)=y_{d}\left(t\right)+y_{s}\left(t\right),$$

where y(t) is the observed (measured) signal at the time *t*, $y_{d}\left(t\right)$ is the true, deterministic part of the signal, and $y_{s}\left(t\right)$ is a stationary noise, stochastic (random) part in the signal, which we assume that it has zero mean.

Let us recall the Gaussian low-pass filter in the time domain, which is defined as the convolution of measured (observed) signal y(t), and the Gauss function ϕ(t;σ) is as follows:

(9) $$y_{Gf}\left(t\right)=y\left(t\right)*\phi \left(t;\sigma \right)=\int_{-\infty }^{\infty }y\left(t-\tau \right)\phi \left(\tau ;\sigma \right)d\tau ,$$

where $y_{Gf}\left(t\right)$ is an output from the filter, i.e., filtered signal.

### 2.4. Proposed Filter

Following the idea of a generalization of the exponential function to the Mittag–Leffler function of two parameters, a novel generalized filter, let us name it the Mittag–Leffler filter, can be defined as follows:

(10) $$y_{MLf}\left(t\right)=y\left(t\right)*\phi \left(t;\sigma ,\alpha ,\beta \right)=\int_{-\infty }^{\infty }y\left(t-\tau \right)\phi \left(\tau ;\sigma ,\alpha ,\beta \right)d\tau .$$

We obtain the classical Gaussian filter (9) for parameters α=1 and β=1. Moreover, we obtain many filters with adjustable forgetting factors with α, β, and σ as tuning knobs. In other words, we can shape the curve of the probability-density function. On the other hand, the different distribution shapes mean that we use different distribution functions, for example, Cauchy distribution, normal distribution, and many others from this family.

Taking into account the Mittag–Leffler distribution (7) and filter definition (10), we can write the novel generalized filter as

(11) $$y_{MLf}\left(t\right)=\frac{1}{\sigma \sqrt{2\pi }}\int_{-\infty }^{\infty }y\left(t-\tau \right)E_{\alpha ,\beta }\left(-\frac{{\left(\tau -\bar{\tau }\right)}^{2}}{2\sigma^{2}}\right)d\tau .$$

Such a three-parameter filter is more flexible than the classical one. It has more degrees of freedom due to the additional tuning parameters α and β, with which we can shape the distribution curve and, therefore, exponential-type forgetting.

### 2.5. Implementation Notes

For the practical implementation of the Gaussian filter in the discrete-time domain, we may use the methods described, for instance, in [27,28]. The Gaussian filter (9) is not causal, meaning the filter window is symmetric in the time domain. It makes the Gaussian filter practically infeasible because the Gaussian function (1) for x∈(−∞,∞) would theoretically require an infinite window length. For practical implementation, it is reasonable to shorten the filter window and use it directly for narrow windows. However, in some cases, this truncation can cause significant errors. In real-time systems, there is a delay because the incoming samples must fill the filter window before the filter can be applied to the signal being processed. The Gaussian filter kernel in the convolution is continuous. The most common replacement for the continuous kernel is the discrete equivalent sampled Gaussian kernel, represented by sampling points from the continuous Gaussian kernel. Instead of an integration operation in convolution, the summation operation over all samples can be used.

It is also well-known that conventional averaging filters based on a moving average, or based on a weighted moving average with exponential forgetting, are not always suitable for their method of assigning weights to older samples of the filtered signal. A frequent request is that the lowest weight was not assigned to the oldest sample but to the sample with a high proportion of the stochastic component. On the other hand, such filters ensure a quick response to a change to a deterministic or stochastic component by assigning a higher weight to more current components. A requirement is also that the filter algorithm was mathematically and, in terms of programming, relatively simple for use even in digital controllers with a limited computing capacity and so that the use of the filter was not limited due to a large number of stochastic values in the measured waveforms.

We may expect similar problems as mentioned above in the Mittag–Leffler filter implementation. Moreover, the Mittag–Leffler function brings some problems with its implementation in real-time applications due to the infinity upper sum limit in the definition. This problem can be circumvented by using the definition of the Mittag–Leffler function in the integral form [14]. However, there can be a problem with the numerical integration method. These limitations were partially solved, and Podlubny and Kacenak proposed a practical implementation algorithm for the Mittag–Leffler function as a Matlab function [29]. For further investigation, we will use it, and its header is:

function [e]=mlf(alpha,beta,Z,P)
%
% MLF(alpha,beta,Z,P) is the Mittag--Leffler function
% E_{alpha,beta}(Z) evaluated with accuracy 10^(-P)
% for each element of Z, alpha and beta are scalars,
% P is integer, Z can be a vector or a 2-dimensional
% array. The output is of the same size as Z.

The aforementioned filter (11) can be easily implemented as a Matlab function using function mlf(alpha,beta,Z,P). The Matlab function of the suggested Mittag–Leffler filter (11) has the following header [30]:

function [y_filt] = ML_filter(t,y,sigma,alpha,beta)
%
% Inputs: t = independent variable, e.g., time
% y = noisy data to be filtered at points t
% sigma = standard deviation
% alpha,beta = parameters of Mittag--Leffler function
% Output:y_filt = filtered data given in variable y

## 3. Results and Discussion

### 3.1. Simulation Examples

For an illustration of the Mittag–Leffler filter benefits, we use the filter (11). The sampling interval is 0.01 in both cases.

In the first example, we compare the Gaussian and Mittag–Leffler filters on the noisy signal given by the function $y_{1}\left(t\right)=e^{-t}\sin\left(3t+1\right)$ noised with random noise from a normal distribution.

**Figure 3:**
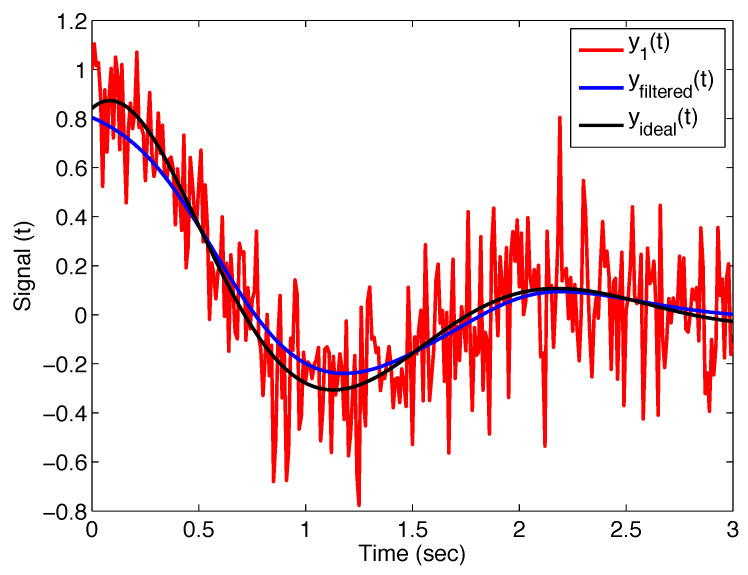
Output of the Mittag–Leffler filter (11) with parameters σ=0.2, α=1, and β=1 (i.e., Gaussian filter) applied on noisy test signal $y_{1}\left(t\right)$, and its comparison with ideal curve.

Figure 3 depicts the noisy signal $y_{1}\left(t\right)$, the ideal curve without noise $y_{ideal}\left(t\right)$, and the filtered signal $y_{filtered}\left(t\right)$ with the filter (11); the filter parameters are σ=0.2, α=1, and β=1. We deal with the classical Gaussian filter (9) for these parameters α and β.

**Figure 4:**
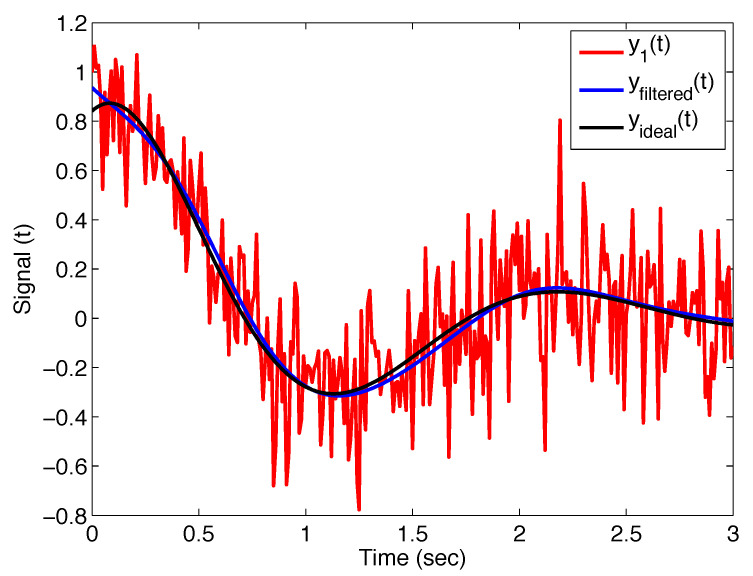
Output of the Mittag–Leffler filter (11) with parameters σ=0.2, α=1.20, and β=1 applied on noise test signal $y_{1}\left(t\right)$, and its comparison with ideal curve.

Figure 4 shows the noisy signal $y_{1}\left(t\right)$, the ideal curve without noise $y_{ideal}\left(t\right)$, and the filtered signal $y_{filtered}\left(t\right)$ with the filter (11); the filter parameters are σ=0.2, α=1.20, and β=1. Thus, we obtain the Mittag–Leffler filter.

In the second example, we compare the Gaussian filter with the Mittag–Leffler filter using the noisy signal given by the function $y_{2}\left(t\right)=\sin\left(\pi t/0.7\right)+\cos\left(2\pi t\right)$ noised with random noise from a normal distribution.

**Figure 5:**
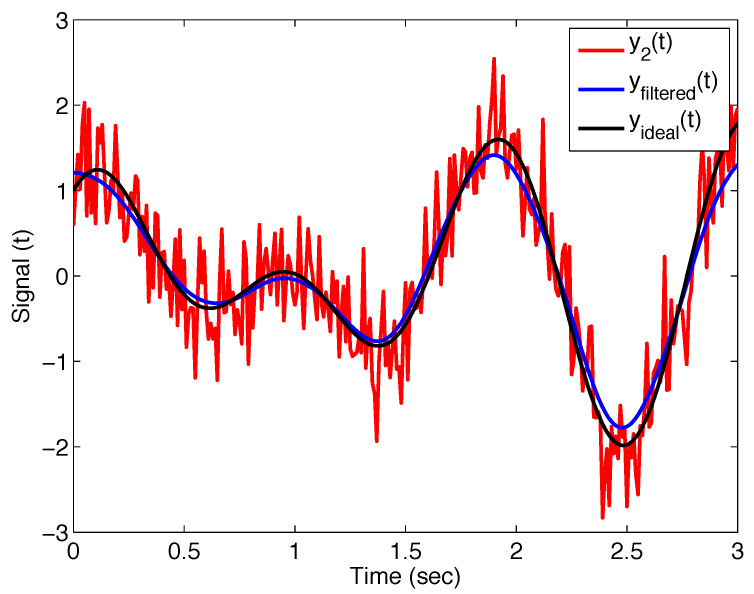
Output of the Mittag–Leffler filter (11) with parameters σ=0.1, α=1, and β=1 (i.e., Gaussian filter) applied on noisy test signal $y_{2}\left(t\right)$, and its comparison with ideal curve.

Figure 5 shows the noisy signal $y_{2}\left(t\right)$, the ideal curve without noise $y_{ideal}\left(t\right)$, and the filtered signal $y_{filtered}\left(t\right)$ with the filter (11); the filter parameters are σ=0.1, α=1, and β=1. Such parameter values for α and β lead to the classical Gaussian filter (9).

**Figure 6:**
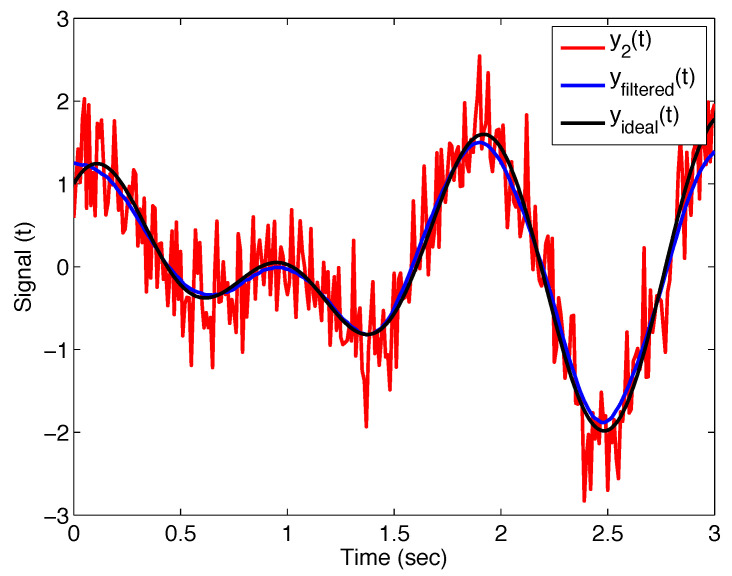
Output of the Mittag–Leffler filter (11) with parameters σ=0.1, α=0.95, and β=0.90 applied on noise test signal $y_{2}\left(t\right)$, and its comparison with ideal curve.

Figure 6 presents the noisy signal $y_{2}\left(t\right)$, the ideal curve without noise $y_{ideal}\left(t\right)$, and the filtered signal $y_{filtered}\left(t\right)$ with the filter (11); the filter parameters are σ=0.1, α=0.95, and β=0.90. Obviously, for these parameters set, we obtain the Mittag–Leffler filter.

Table 1 summarizes the comparison of the mean squared errors (MSE) for two test signals and different sets of filter parameters, which were experimentally found via a simulation approach and presented above.

### 3.2. Real ECG Signal

In additional examples, we will use real ECG signals of the heart’s electrical activity. It should be noted that arrhythmia analysis and related subjects were studied, and one of the first significant product of that effort was the MIT-BIH arrhythmia database [31]. The sampling frequency was 360 ticks per second in both cases. These signals are noised, and the presented examples show how to use a generalized low-pass filter in the signals, which contain low-amplitude high-frequency noise.

Firstly, record number 122 (male, age 51, used medications: Digoxin, Lasix, and Pronestyl) was chosen from the mentioned database [31].

**Figure 7:**
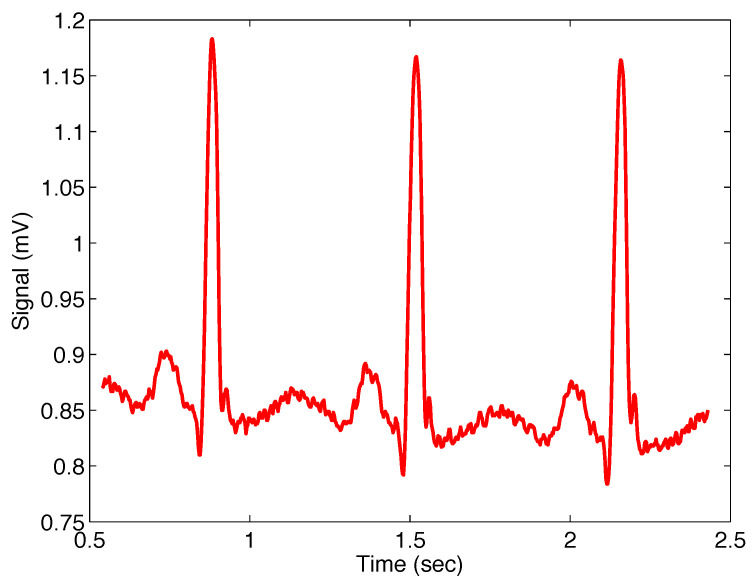
Selected sequence of real ECG signal, sample number 122, from database [31].

Figure 7 presents the noisy real ECG signal within approximately 0.5 to 2.5 s.

**Figure 8:**
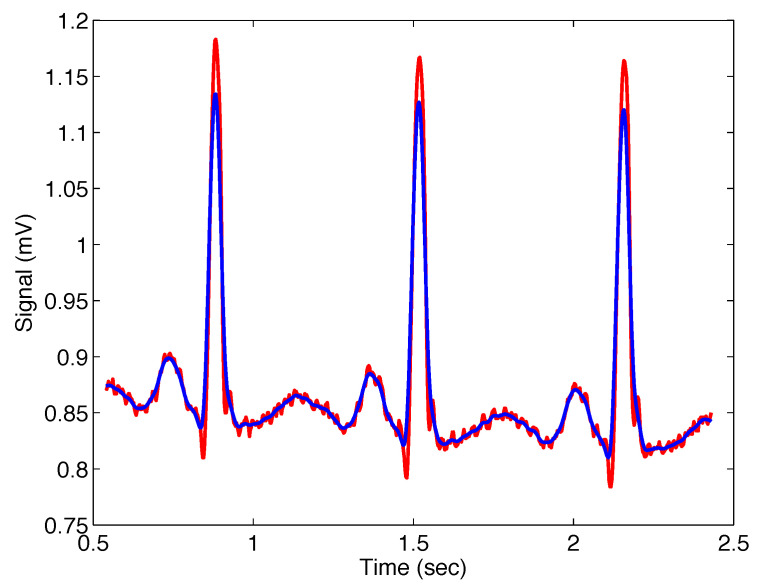
Output from the Mittag–Leffler filter (11) (blue) with parameters σ=0.01, α=1, and β=1 (i.e., Gaussian filter), applied on noisy ECG signal (red).

Figure 8 presents the noisy real ECG signal and the filtered signal with the filter (11); the filter parameters are σ=0.01, α=1, and β=1, which means that the Gaussian filter was applied.

**Figure 9:**
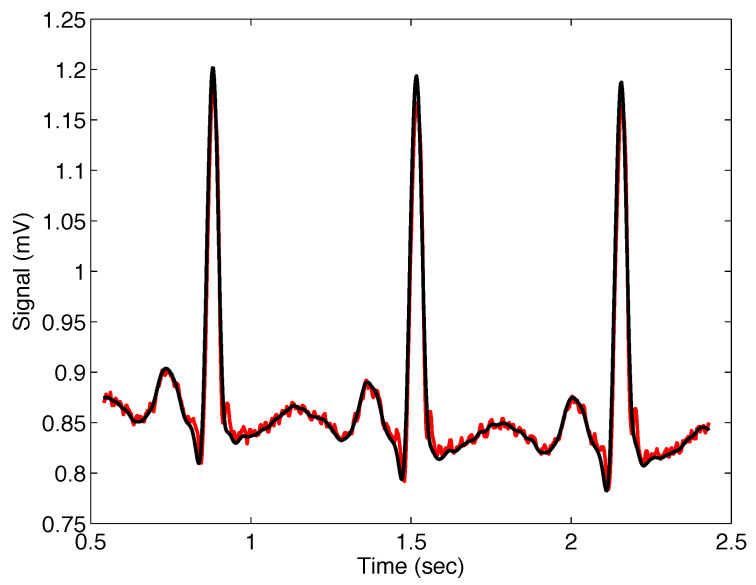
Output from the Mittag–Leffler filter (11) (black) with parameters σ=0.01, α=1.20, and β=1, applied on noisy ECG signal (red).

Figure 9 presents the noisy real ECG signal and the filtered signal with the Mittag–Leffler filter (11); the filter parameters are σ=0.01, α=1.20, and β=1. These parameters were found experimentally via a simulation approach.

Secondly, record number 232 (female, age 76, used medications: Aldomet, Inderal) was chosen from the database as mentioned earlier [31].

**Figure 10:**
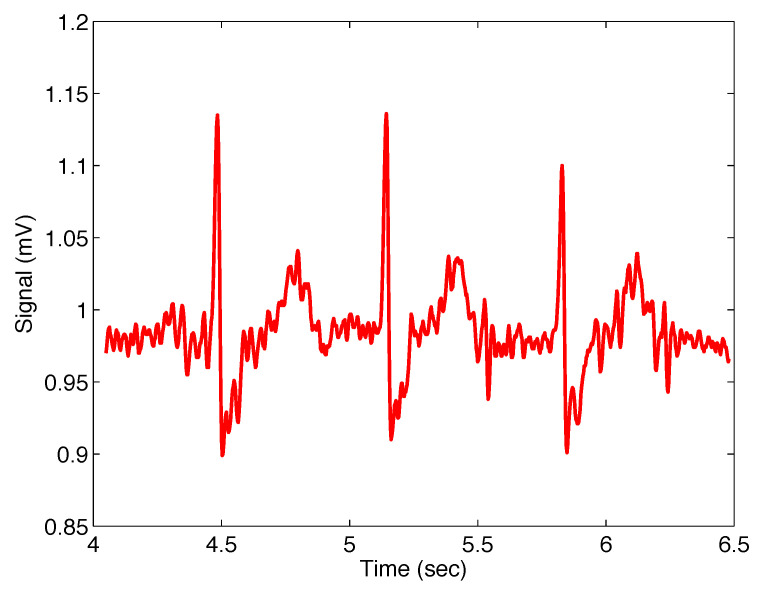
Selected sequence of real ECG signal, sample number 232, from database [31].

Figure 10 presents the noisy real ECG signal within approximately 4 to 6.5 s.

**Figure 11:**
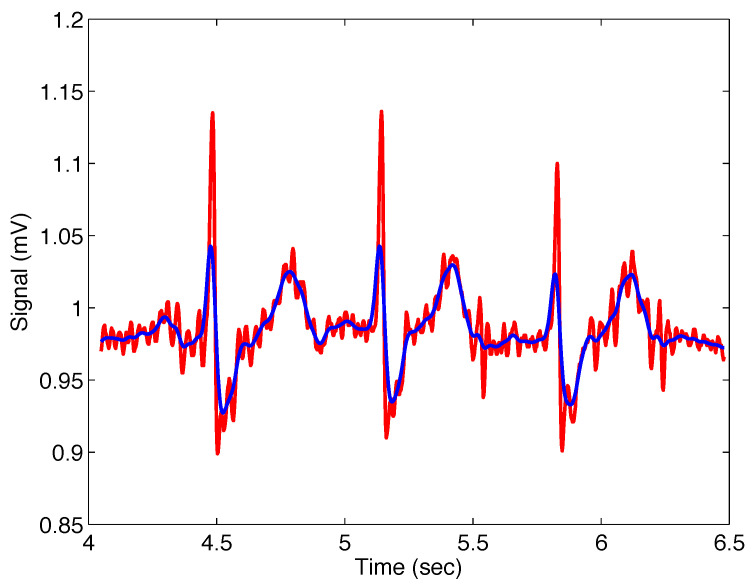
Output from the Mittag–Leffler filter (11) (blue) with parameters σ=0.015, α=1, and β=1 (i.e., Gaussian filter), applied on noisy ECG signal (red).

Figure 11 presents the noisy real ECG signal and the filtered signal with the filter (11); the filter parameters are σ=0.015, α=1, and β=1, which means that the Gaussian filter was applied.

**Figure 12:**
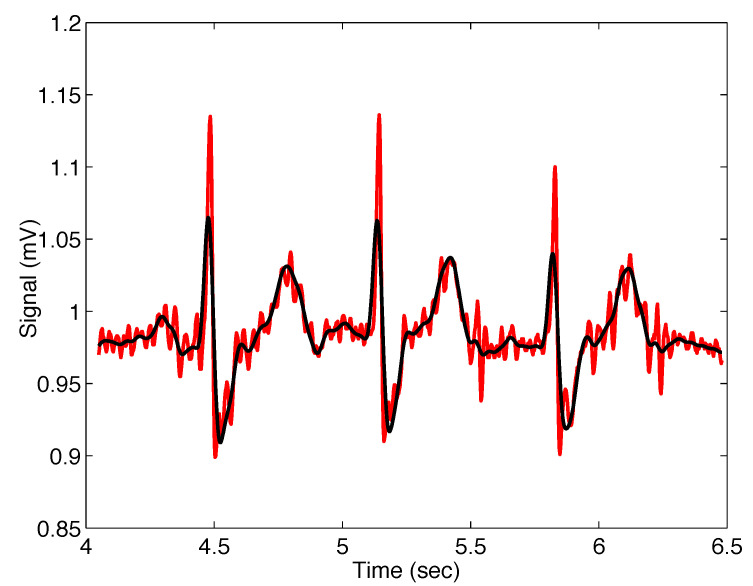
Output from the Mittag–Leffler filter (11) (black) with parameters σ=0.015, α=1.20, and β=1, applied on noisy ECG signal (red).

Figure 12 presents the noisy real ECG signal and the signal filtered using the Mittag–Leffler filter (11); the filter parameters are σ=0.015, α=1.20, and β=1, where the same approach was used for the setting as in the previous case.

Table 2 summarizes the filter performance comparison of the mean squared errors (MSE) as the measure, calculated from filtered ECG and the original noisy ECG signals, for two subjects and different sets of filter parameters found (experimentally) via a simulation approach.

It can be observed from the peaks and those parts where the noise is not so significant, as well as results presented in Table 2, that the new Mittag–Leffler filter again provides better results than the classical Gaussian filter. Moreover, the major waves P and T and the others Q, R, and S can be reliably identified, read, and interpreted. However, the F-waves in the atrial fibrillation segment could be smoother. Different filter parameters or denoising methods should be used to solve this problem [32].

As we can see from these first results, the Mittag–Leffler filter gives better results than the Gaussian filter due to more adjustable parameters. It means that we can shape the distribution function’s curve and thus the exponential-type forgetting factor. That is the main benefit of the novel generalized filter, which is helpful in advanced ECG signal-processing. Other filters, such as the Butterworth filter or the Savitzky–Golay filter, can also be used for ECG denoising. A comparison of ECG signals from the same MIT-BIH database with different sampling frequencies and filters, which improve the quality of ECG signals, can be found in [33].

## 4. Conclusions

This paper presents a novel generalized low-pass filter based on the Mittag–Leffler function for signal processing applications. The proposed filter has three adjustable parameters and is more flexible than the classical Gaussian filter. For a specific set of filter parameters, the Gaussian filter is a particular case of the new Mittag–Leffler filter. A Matlab function of the suggested Mittag–Leffler filter was created as well. Two simulation examples present the benefits of the Mittag–Leffler filter by comparing the values of the MSE with the Gaussian filter. Additional examples, real measured ECG signals, confirm that the first theoretical results are helpful even in such a significant area of application as ECG-signal denoising. Moreover, the introduced Mittag–Leffler filter presents new possibilities for applications in denoising other medical signals, including EEG and EMG.

Methods, tools, and techniques proposed and used in this paper allow further extension of the proposed Mittag–Leffler filter into two dimensions for image-processing applications.

## Figures and Tables

**Table 1:** Mean squared errors comparison.

Figure	Signal	Filter (11) Parameters (σ, α, β)	MSE Value
#3	$y_{1}\left(t\right)$	σ=0.2, α=1.00, β=1.00	0.0027
#4	$y_{1}\left(t\right)$	σ=0.2, α=1.20, β=1.00	0.0019
#5	$y_{2}\left(t\right)$	σ=0.1, α=1.00, β=1.00	0.0233
#6	$y_{2}\left(t\right)$	σ=0.1, α=0.95, β=0.90	0.0138

**Table 2:** Mean squared errors comparison.

Figure	Signal	Filter (11) Parameters (σ, α, β)	MSE Value
#8	ECG #122	σ=0.01, α=1.00, β=1.00	202.8265
#9	ECG #122	σ=0.01, α=1.20, β=1.00	127.2508
#11	ECG #232	σ=0.015, α=1.00, β=1.00	136.0610
#12	ECG #232	σ=0.015, α=1.20, β=1.00	121.2894

## Data Availability

Not applicable.

## Abbreviations

The following abbreviations are used in this manuscript:

EEG	Electroencephalographic
EMG	Electromyographic
ECG	Electrocardiographic
PDF	Probability-density function
Gf	Gaussian filter
MLf	Mittag–Leffler filter
